# Long-term methionine-diet induced mild hyperhomocysteinemia associated cardiac metabolic dysfunction in multiparous rats

**DOI:** 10.14814/phy2.12292

**Published:** 2015-05-25

**Authors:** Su Song, Elizabeth Kertowidjojo, Caroline Ojaimi, Beatriz Martin-Fernandez, Sharath Kandhi, Michael Wolin, Thomas H Hintze

**Affiliations:** Department of Physiology, New York Medical CollegeValhalla, New York, 10595, USA

**Keywords:** Mild hyperhomocysteinemia, multiparous, NAD(P)H oxidase, nitric oxide, superoxide

## Abstract

Mild hyperhomocysteinemia (HHcy, clinically defined as less than 30 *μ*mol/L) is an independent cardiovascular disease (CVD) risk factor, and is associated with many complications during pregnancy, such as preeclampsia (PE). The aim of this study was to assess the effect of long-term mild HHcy on cardiac metabolic function of multiparous rats. Female rats were mated 3 to 4 times and were fed with methionine in drinking water to increase plasma Hcy (2.9 ± 0.3 to 10.5 ± 2.3 μmol/L) until termination. This caused significant increase of heart weight/body weight (0.24 ± 0.01 to 0.27 ± 0.01 g/100 g) and left ventricle weight (0.69 ± 0.03 to 0.78 ± 0.01 g). Superoxide production was increased by 2.5-fold in HHcy hearts using lucigenin chemiluminescence. The ability of bradykinin and carbachol to regulate myocardial oxygen consumption (MVO_2_) in vitro was impaired by 59% and 66% in HHcy heart, and it was restored by ascorbic acid (AA), tempol, or apocynin (Apo). Protein expression of p22^phox^ subunit of NAD(P)H oxidase was increased by 2.6-fold, but there were no changes in other NAD(P)H oxidase subunits, NOSs or SODs. Microarray revealed 1518 genes to be differentially regulated (*P *<* *0.05). The mRNA level of NAD(P)H oxidase subunits, NOSs or SODs remained unchanged. In conclusion, long-term mild HHcy increases cardiac superoxide mainly through regulation of p22^phox^ component of the NAD(P)H oxidase and impairs the ability of NO to regulate MVO_2_ in heart of multiparous mothers.

## Introduction

Hyperhomocysteinemia (HHcy) is common in women, and can be induced for genetic or nutritional reasons, such as deficiency in cystathionine-*β*-synthase (CBS), methylenetetrahydrofolate reductase (MTHFR), vitamin, or folic acid (Perry 1999). Over the past decades, mild HHcy, defined clinically as less than 30 *μ*mol/L, has been recognized as an independent risk factor for a variety of cardiovascular diseases (CVD) including coronary artery disease, peripheral arterial disease and chronic heart failure (Perry 1999; Zylberstein et al. 2004; Vizzardi et al. 2009).

Clinical evidence has shown that a number of complications of pregnancy, such as preeclampsia (PE) and intrauterine growth restriction (IUGR) have multiple causes, are also associated with elevated plasma homocysteine (Hcy) (Lopez-Quesada et al. 2003; Lindblad et al. 2005). Women with a history of these complications are at higher risk to repeat in subsequent pregnancies and higher risk of CVD in later life (Varvarigou 2010; Duckitt and Harrington 2005; Bellamy et al. 2007). Given the well documented correlation between mild HHcy (~ 10 *μ*mol/L) and CVD, we hypothesize that mild HHcy contributes to the cardiac dysfunction in pregnancy-related complications such as PE, and may account for the increased risk of reoccurrence and CVD in later life.

Nitric oxide (NO) plays an important role in regulating: cardiac metabolic function; oxygen consumption; and substrate use during pregnancy (Williams et al. 2007). Reduced bioavailability of NO is thought to be one of the central factors common to myocardial ischemia, atherosclerotic disease, and heart failure (Cannon 1998; Trochu et al. 2003). NO bioavailability has been reported to be impaired by mild HHcy through increased oxidative stress, and one major source of the oxidative stress in cardiovascular system is superoxide (Kolling et al. 2011; Ungvari et al. 2003; Becker et al. 2005; Edirimanne et al. 2007; Suematsu et al. 2007). A more recent study has also suggested a link between NO inactivation and pregnancy-related disease, by showing that serum NO metabolites concentration is decreased in preeclamptic patients and endothelial cells. NAD(P)H oxidase subunit gp91^phox^ expression is increased by treatment with sera from preeclamptic patients (Matsubara et al. 2010). The possible long-term effect of mild HHcy on maternal heart with multiparity has received little attention. Therefore, the aim of this study was to assess the effect of long-term mild HHcy on cardiac metabolic function in multiparous rats, and to explore the possible role of NO and superoxide in pathogenesis.

## Material and Methods

### Animals and material preparations

Female Sprague–Dawley (SD) Rats (200–250 g) were purchased from Charles River Laboratories. Control male SD rats (250 g) were used for mating; each male was housed with two females in a new, clean cage. Female rats were pregnant 3–4 times. For the first two pregnancies, newborns were taken away immediately after birth. For the third or fourth pregnancy, after confirmation of pregnancy (Day 1; determined by presence of a plug on the morning after mating), the females were immediately removed from the males, and placed into new cages for the duration of the experiment. Drinking water with or without l-methonine (Sigma-Aldrich, 9 g/L) was supplied from day one of first pregnancy till sacrifice.

Animals were killed with sodium pentobarbital (50 mg/kg, ip) on day 19–21 of 3rd or 4th pregnancy. Blood was collected from the left ventricle (LV). The hearts were immediately harvested and weighed. Some fresh LV tissues were used for oxygen consumption and superoxide measurement. The rest of the tissues and plasma were immediately frozen in liquid nitrogen and were stored in −80°C until use.

The protocol of the study was approved by the Institutional Animal Care and Use Committee of New York Medical College and followed the current guidelines of the National Institutes of Health and American Physiological Society for the use and care of laboratory animals.

### Measurement of plasma total Hcy

Plasma total Hcy (tHcy) was measured using a microplate enzyme immunoassay (Bio-Rad Laboratories), and the signal was read at 450 nm by a Power Wave 200 spectrophotometer (Bio-Tek) as described previously (Becker et al. 2005).

### Hemodynamic and cardiac structural measurements

At day 18/19 of the 3rd or 4th pregnancy, a noninvasive blood pressure monitor (NIBP-8, Columbus Instruments) was used to assess conscious resting heart rate (HR) (Table1) and blood pressure and cardiac structure was measure by transthoracic echocardiography performed with a 15-MHz linear transducer (Acuson) as described previously (Becker et al. 2005). Cardiac output (CO) was calculated as the product of stroke volume (SV) and heart rate (measured during echocardiography) and total peripheral resistance (TPR) was calculated as mean arterial pressure (MAP) divided by cardiac output.

**Table 1:** Hemodynamic data

	Control multiparous pregnant rats (*n* = 6)	HHcy multiparous pregnant rats (*n* = 6)
Weight at time of death, g	433.7 ± 4.8	422.8.3 ± 12.7
Heart weight, g	1.03 ± 0.01	1.14 ± 0.01^1^
Heart weight/body weight, %	0.24 ± 0.01	0.27 ± 0.01^1^
Left Ventricle weight, g	0.69 ± 0.03	0.78 ± 0.01^1^
Septum weight, g	0.19 ± 0.01	0.18 ± 0.01
Uterine weight, g	17.89 ± 1.08	14.58 ± 0.86^1^
Total Fetal weight, g	48.1 ± 16.5	34.0 ± 10.7^1^
Number of pups	15.6 ± 0.92	16.7 ± 0.97

	*n* = 10	*n* = 9
Blood pressure monitor		
SBP, mmHg	114.9 ± 4.0	139.6 ± 4.4^1^
DBP, mmHg	89.2 ± 2.3	94.3 ± 4.3
MBP, mmHg	97.0 ± 2.8	108.0 ± 4.1^1^
Stroke volume, mL	0.39 ± 0.03	0.31 ± 0.02
Heart rate, conscious, bpm	446.1 ± 33.5	411.7 ± 21.9
Cardiac output, mLmin^−1^	167.7 ± 12.5	132.0 ± 9.6^1^
Total peripheral resistance, mmHgmin^−1 ^m^−1^	0.64 ± 0.09	0.86 ± 0.20^1^

1 Versus control *P *<* *0.05.

### In vitro myocardial oxygen consumption measurement

Myocardial oxygen consumption (MVO_2_) was measured and the role of NO determined as previously described (Adler et al. 2003). Cumulative doses of bradykinin (BK) or carbachol (Cch) (10^−7^ to 10^−4^ mol/L) were added to stimulate NO production in vitro. The effects of 10^−3^ mol/L superoxide scavenger ascorbic acid (AA), 10^−4^ mol/L NAD(P)H oxidase inhibitor apocynin (Apo), 10^−3^ mol/L SOD mimetic tempol, or 10^−3^ mol/L NOS inhibitor L-NAME on MVO_2_ were expressed as percent change of baseline.

### Lucigenin-enhanced superoxide measurement

Superoxide generation detected by lucigenin-enhanced chemiluminescence was measured in a liquid scintillation counter (Beckman LS-6000IC) as previously described (Iesaki et al. 1999). 10^−3^ mol/L apocynin, 10^−3^ mol/L tiron, or 10^−3^ mol/L ascorbic acid were also used.

### NOx measurement

The NO end products nitrate and nitrate were measured by a Nitrate/Nitrite Colorimetric Assay Kit (Cayman Chemical) following the protocol provided with the kit. A total of 25 *μ*mol/L EDTA-plasma were used and the absorbance was measured at 550 nm.

### Western blot

The preparation of protein samples from myocardial tissues was performed as previously described (Becker et al. 2005). Antibodies to eNOS (BD Transduction Laboratories, 1:250 dilution), phospho-eNOS (Ser 1177) (Cell Signal, 1:500 dilution), SOD-1(Calbiochem, 1:5000 dilution), SOD-2 (BD Transduction Laboratories, 1:10,000 dilution), SOD-3 (Santa Cruz Biotechnology, 1:5000 dilution), or one of the following subunits of NAD(P)H oxidase: p67^phox^ (Upstate, 1:1000 dilution), p22^phox^ (Santa Cruz Biotechnology, 1:2000 dilution), gp91^phox^(BD Transduction Laboratories, 1:1000 dilution), p47^phox^ (Santa Cruz Biotechnology, 1:1000 dilution), phos-p47^phox^ (Upstate, 1:1000 dilution), p40^phox^ (Santa Cruz Biotechnology, 1:800 dilution), Rac-1 (Santa Cruz Biotechnology, 1:5000 dilution), and nitrotyrosine (Santa Cruz Biotechnology, 1:1000 dilution) were used.

### RNA isolation and microarray analysis

Total RNA was extracted from the control (*n *=* *4) and Met-fed (*n* = 4) LV tissues as previously described (Ojaimi et al. 2005). RNA quality was assessed by electrophoresis with the Agilent Bioanalyzer 2100 (Agilent Technologies, Santa Clara, Calif). cDNA was hybridized to Affymetrix GeneChip Rat Genome 230 2.0 array. Determination of statistical significance for changes in gene expression was performed in GeneSpring with a *t*-test and with variance stabilization.

### Statistical analysis

All data were presented as mean and SEM. A statistical significance of differences was determined with t-test. Changes are considered significant at *P *<* *0.05.

## Results

### Measurement of total plasma Hcy

Total plasma Hcy level was significantly increased threefold in methionine-fed animals (10.5 ± 2.3 *μ*mol/L) as compared to control animals (2.9 ± 0.3 *μ*mol/L) (*P *<* *0.05).

### Hemodynamic measurements

All hemodynamic data and weights are shown in Table1. Heart weight (HW) and LV weight were significantly increased by 11% and 13% in HHcy rats as compared to control. When HW is normalized by body weight, there was also a significant increase in HHcy group, indicating cardiac hypertrophy.

Average systolic blood pressure increased from 114.9 ± 4.0 mmHg of control to 139.6 ± 4.4 mmHg. In the HHcy group, six of nine rats developed hypertension (SBP>140 mmHg). TPR was more than 30% higher in HHcy rats compared to control, suggesting an impairment of peripheral endothelial function.

High concentrations of plasma tHcy have been associated with a greater risk of IUGR in human (Pierantognetti et al. 2003). We (data unpublished) and others have previously found that methionine-diet-induced mild HHcy has a negative effect on fetal growth (Kassab et al. 2005). Consistent with those findings, in this study, while the number of offspring was similar between groups, the fetal weight was significantly smaller in HHcy group than control, suggesting an intrauterine growth restriction.

### Superoxide measurements

**Figure 1:**
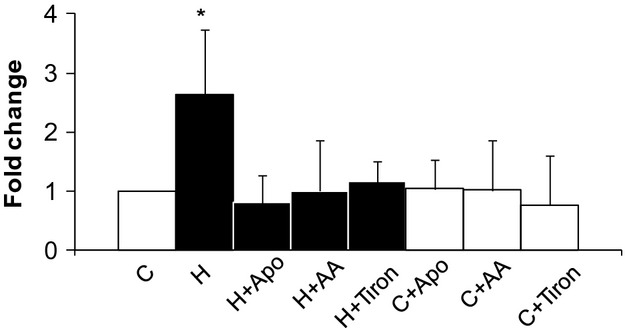
Superoxide detected by Lucigenin-enhanced chemiluminescence. Superoxide was significantly increased in HHcy hearts (H, black bar) as compared to control (C, open bar), and coincubation with AA (10^−3^ mol/L), tiron (10^−3^ mol/L), or Apo (10^−3^ mol/L) abolished the effect. *n* = 6 each group; *versus control ≤ 0.05.

Increased oxidative stress has long been linked to mild HHcy pathogenesis. Previously we have found that superoxide was elevated in male dog and rat hearts (Becker et al. 2005; Suematsu et al. 2007) given methionine. In this study, we measured superoxide by lucigenin-enhanced chemiluminescence, and found a 2.6 ± 1.1-fold increase of signal in HHcy hearts as compared to control (Fig.1). AA or tiron were incubated with the tissues and each abolished the increased signal in HHcy heart, confirming the elevated signal was from increased superoxide production. NAD(P)H oxidase has been the most likely source of superoxide in cardiac tissue, and it is activated by mild HHcy in different species and tissues (Ungvari et al. 2003; Becker et al. 2005; Suematsu et al. 2007). So we hypothesized that the increased superoxide in the heart of multiparous mothers with mild HHcy was mainly derived from NAD(P)H oxidase. To test this, we incubated HHcy LV tissues with NAD(P)H oxidase inhibitor apocynin. As expected, there was a suppression of the signal similar to when AA or tiron were used. So far, we have demonstrated that mild HHcy-induced NAD(P)H oxidase-derived superoxide in multiparous rats hearts.

### Effect of Hcy on MVO_2_ in tissues

NO rapidly reacts with superoxide at an almost diffusion-limited rate (Miles et al. 1996). It has been shown that change in NO and superoxide is always negatively correlated (Ungvari et al. 2003; Terman et al. 2004). We considered that the increase in superoxide would result in a decrease in NO bioactivity. It has been well established that NO inhibits cardiomyocyte oxygen consumption by binding to mitochondria cytochrome c oxidase, and the bioavailability of BK-stimulated endogenous NO can be experimentally measured (Wainio 1955; Carr and Ferguson 1990; Poderoso et al. 1998; Loke et al. 1999). In this study, we examined the ability of BK-induced NO to regulate MVO_2_.

**Figure 2:**
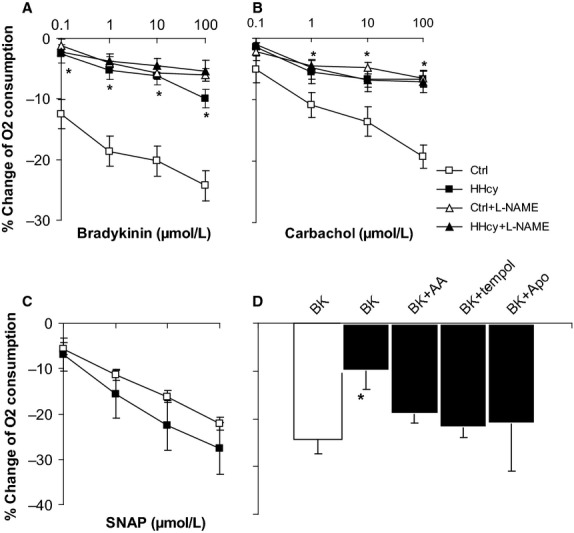
Changes in MVO_2_ in LV tissues. BK (bradykinin) (A) was used to stimulate the endogenous NO release. In control rats (♦), O_2_ consumption was inhibited in a dose-dependent manner in response to accumulated doses of BK, and these effects were significantly attenuated in HHcy rats (■). Adding L-NAME in tissue bath attenuated the response in control rats (∆), but did not affect HHcy rats (▲) (*n* = 6 each); B, Cch had similar effect as BK; C, exogenous NO donor SNAP had similar effect on both groups; D, The ability of BK (10^−4^) to reduce MVO_2_ in mild HHcy group was restored after coincubation with AA (ascorbic acid) (10^−3^ mol/L), tempol (10^−3^ mol/L), or Apo (apocynin) (10^−3^ mol/L);

BK stimulates eNOS causing the endogenous release of NO. As shown in Figure2, there was a dose-dependent decrease in MVO_2_ in both groups with cumulative doses of BK (10^−8^ to 10^−4^ mol/L) (Fig.2). Cch stimulates NO production from eNOS through a different receptor (M1) and was used here to reinforce the effect of BK on eNOS. We found that the responses to Cch (10^−7^ to 10^−4^ mol/L) are similar to those of BK. As compared to control, the response was significantly suppressed in HHcy hearts, and the degree of suppression was of the same magnitude as L-NAME treated control tissues. In addition, L-NAME treatment did not shift the response curve any further to the right. These findings suggested that cardiac NO bioavailability is impaired by mild HHcy. Similar to L-NAME treated tissues, the response to BK and Cch in HHcy hearts were significantly suppressed (10.0 ± 3.9% and 7.2 ± 0.6%) (*P *<* *0.05, *n* = 6) as compared to control, and further, adding L-NAME into the tissue bath did not cause any additional significant change. This suggests that NO bioavailability was markedly impaired in HHcy hearts. When NO donor SNAP was used, HHcy hearts responded similarly as control hearts, suggesting that mild HHcy did not impair the tissue sensitivity to NO.

### Effect of antioxidants on regulation of oxygen consumption

While the presence of antioxidants did not significantly alter MVO_2_ in control hearts in response to BK, the suppressed response in HHcy hearts was completely restored by co-incubation with ascorbic acid (19.2 ± 1.8%) and SOD mimetic tempol (21.9 ± 2.1%), suggesting that NO was impaired by increased superoxide. The ability of NAD(P)H oxidase inhibitor apocynin to restore the MVO_2_ in HHcy hearts again suggested that NAD(P)H oxidase is the major source of superoxide (Fig.2).

### NOx measurement

NOx (nitrate and nitrite) are the end products of NO metabolites, and reflect the endogenous production of NO. HHcy mothers had a similar level of plasma NOx (21.9 ± 0.2 *μ*mol/L) as compared to control mothers (23.5 ± 0.5 *μ*mol/L), suggesting that mild HHcy did not increase NO production.

### Western blot analysis

**Figure 3:**
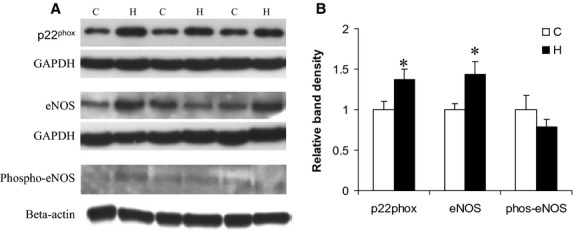
Western blot showed increased protein expression of p22^hpox^ subunit and eNOS. A) Representative western blots of p22^phox^, eNOS and phosphor-eNOS from control hearts (C) and HHcy hearts (H). B) Summary data for the density analysis of p22^phox^, eNOS and phosphor-eNOS. *versus control *P *≤* *0.05

Protein expression of NAD(P)H oxidase subunits was measured by Western blot. After normalized by GAPDH, the p22^phox^ subunit was significantly increased by 37% (*P *<* *0.05) (Fig.3). Nitrotyrosine was similar between groups. We also measured SOD-1, SOD-2 and SOD-3, and found no difference between groups. eNOS expression was 43% increased in HHcy hearts as compared to control (Fig.3), but phospho-eNOS was not changed.

### Microarray analysis

Of the 31,099 transcripts and variants on the microarrays, a total of 1518 genes in HHcy animals were found differentially regulated (*P *<* *0.05) as compared to control. Among them, 265 genes were more than ±1.5-fold changed, with 199 upregulated and 66 downregulated.

Table2 listed some interesting genes that were significantly changed in HHcy animals. (All hybridization data have been submitted to the National Center for Biotechnology Information (NCBI) Gene Expression Omnibus database with GEO Accession Numbers for series GSE30308.) At the transcriptional level, mRNA of NAD(P)H oxidase subunits was not changed. Most of the antioxidants and antioxidant enzymes genes, such as SODs, and glutathionine reductase, were not significantly changed. On the basis of their GO biological process annotations, we grouped the differentially expressed genes into nine functional categories as Cell Cycle/Proliferation, Development/Reproduction, Immune Response/Apoptosis, Metabolism, Protein/Protein Expression, Cell Signaling/Communication, Cell Structure/Motility, Proteolysis, and Unknown/Others. Among them, cell signaling/communication category has the largest number of genes changed (14%). For example, cAMP responsive element modulator (CREM) was downregulated by 1.9-fold, and G-protein-coupled receptor 34 and RAS-like family 11 were upregulated by 2.1- and 1.8-fold. Some structural genes such as tropomyosin 1 and activity-regulated cytoskeleton-associated protein were also differently regulated. Unfortunately, a large portion of the genes that were significantly up or downregulated encode for proteins whose identities have not been well defined.

**Table 2:** Example genes that are significantly changed in HHcy group as compared to control

Probe set ID	Gene title	Fold change HHcy versus Ctrl, *P *<* *0.05
1388395_at	G0/G1switch 2	2.9
1368344_at	Glutamate decarboxylase 1	2.3
1387203_at	Glucokinase regulatory protein	2.2
1377702_at	Purinergic receptor P2Y, G-protein-coupled, 5	2.2
1370747_at	Fibroblast growth factor 9	2.0
1369043_at	Potassium voltage-gated channel	2.0
1393682_at	G-protein-coupled receptor 34	2.1
1378925_at	cAMP responsive element modulator	1.9
1393335_at	EGF-like-domain, multiple 6	1.9
1391384_at	Tumor necrosis factor	1.7
1368983_at	Heparin-binding EGF-like growth factor	1.6
1369771_at	Insulin receptor substrate 1	1.6
1395794_at	Tropomyosin 1, alpha	1.5
1373085_at	Carbonyl reductase 3	1.5
1389474_at	Myosin regulatory light chain interacting protein	1.5
1368172_a_at	Lysyl oxidase	1.4
1387088_at	Galanin prepropeptide	−6.7
1369067_at	Nuclear receptor subfamily 4	−4.9
1387795_at	Polymerase (DNA directed), alpha 2	−1.9
1368188_at	4-hydroxyphenylpyruvate dioxygenase	−1.8
1397782_at	Oxoglutarate dehydrogenase (lipoamide)	−1.7
1370650_s_at	Bradykinin receptor B2	−1.5
1375900_at	Tumor necrosis factor receptor superfamily, member 9	−1.5
1387068_at	Activity-regulated cytoskeleton-associated protein	−1.5
1371077_at	5-hydroxytryptamine (serotonin) receptor 3b	−1.4
1377314_at	Glutamine fructose-6-phosphate transaminase 1	−1.4

## Discussion

The major findings of this study include: (1) mild HHcy caused hypertension in multiparous rats; (2) NAD(P)H oxidase-derived superoxide production was significantly increased without an apparent increase in antioxidants and antioxidant enzymes; (3) increased superoxide attenuated the ability of BK or Cch to regulate MVO_2_ in vitro, and this effect was restored by antioxidant treatment; and (4) cardiac gene expression profile differed between HHcy and control groups.

Increased tHcy has been associated with an increased risk for PE that featured as elevated blood pressure (Khosrowbeygi and Ahmadvand 2013). Although PE is more frequently seen in nulliparous women than in multiparous women, the risk of women who have PE in a first pregnancy is significantly higher in later pregnancies (Duckitt and Harrington 2005; Brown et al. 2007; Hernandez-Diaz et al. 2009). In our study, with mild HHcy starting from the first pregnancy (data not shown) and continuing until termination, overt hypertension occurred in six of nine HHcy multiparous rats, but none in control group developed hypertension. This suggests that HHcy can be a causal factor increasing the risk of PE in multiparous women with a history of PE. Experimental and clinical evidence support a correlation between plasma tHcy level and blood pressure (Brett et al. 2006; Khosrowbeygi and Ahmadvand 2013). Although some other experimental findings question the hypothesis that HHcy is a contributor to the elevation of blood pressure in PE (Chandler et al. 2009) in nulliparous rats, the possibility that the duration of exposure is an important determinant for hypertension to be induced by mild HHcy cannot be excluded. Increased oxidative stress leads to endothelial cells impairment. This is also supported by the findings that HHcy increases oxidative stress and impairs endothelial function. We also measured plasma angiotensin II level, but found no difference between groups (data not shown). It thus is more likely that the increased blood pressure is secondary to elevated oxidative stress.

As far as we know, the effect of mild HHcy on cardiac metabolic function in multiparous mothers has not been studied. Mild HHcy has been demonstrated to increase oxidative stress in many different organs such as brain, lung, kidney, blood vessel, and heart (Ungvari et al. 2003; Becker et al. 2005; Sachdev 2005; Yi et al. 2006; Suematsu et al. 2007; da Cunha et al. 2011). The increase in oxidative stress has been suggested as a link between HHcy and many CVD. We have reported that superoxide is elevated by HHcy in male rat heart and dog heart and our unpublished data on nulliparous rat heart showed the same change (Becker et al. 2005; Suematsu et al. 2007). In this study we found that superoxide was significantly elevated by mild HHcy in multiparous hearts as compared to the controls. As compared with xanthine oxidase, arachidonic acid, and mitochondrial oxidases, NAD(P)H oxidase has been demonstrated as the major source of ROS in cardiac cells (Mohazzab et al. 1997). Previously we have found that in male rat and dog heart and nulliparous rat heart the protein level of NAD(P)H oxidase is elevated by HHcy and by blocking the oxidase the increased superoxide level is restored to control level, suggesting NAD(P)H oxidase is a major source of elevated superoxide in HHcy maternal heart (Becker et al. 2005). In this study, after apocynin blocks NAD(P)H oxidase the increase in superoxide in cardiac tissue was abolished, demonstrating that mild HHcy also increases cardiac oxidative stress through NAD(P)H oxidase-derived superoxide in pregnant rat heart. In fact, the evidence of HHcy increasing oxidative stress through NAD(P)H oxidase-derived superoxide is not limited to cardiac tissue, but was also seen in coronary arteries(Ungvari et al. 2003), vascular smooth muscle, neutrophils, and endothelium (Zou et al. 2010; Bellamy et al. 1998; Alvarez-Maqueda et al. 2004).

The site of NAD(P)H oxidase regulation differs by treatments and disease. For example, in heart failure patient, the p47^phox^ translocation from cytosol to membrane is increased (Heymes et al. 2003). Angiotensin II-induced hypertensive rats have elevated p22^phox^ mRNA in aorta (Fukui et al. 1997); protein expression of p22^phox^, p47^phox^, and p67^phox^ is increased in placentas from PE patients (Dechend et al. 2003); HHcy increases protein expression of p22^phox^ in male rat heart and protein expression of Nox 2 in male dog heart (Becker et al. 2005; Suematsu et al. 2007). Unfortunately, so far the underlying pattern that determines which subunit(s) of the enzyme is regulated and whether the regulation occurs at transcriptional, translational or posttranslational level under different circumstances, has yet to be found. While the degree of phosphorylation of p47^phox^ subunit is enhanced in the heart of nulliparous mothers with mild HHcy (our data), we found an upregulation of p22^phox^ protein but not mRNA in the HHcy heart of multiparous mothers. The mechanisms of cardiac NAD(P)H oxidase activation in short-term HHcy exposure with single pregnancy may differ from long-term exposure with multiple pregnancies, and this might reflect a differentially regulated mechanism which contributes to the increased risk of CVD development in the future.

We further examined whether long-term mild HHcy has affected the antioxidant system in the multiparous heart. Our microarray showed no significant change in antioxidants and antioxidant enzymes genes, such as xanthine dehydrogenase, thioredoxin reductase, glutathione peroxidase, and SOD isoforms. Protein level of SODs was also not different. It seems that the expression of major antioxidant enzymes and antioxidants were not significantly affected. However, we cannot rule out the involvement of antioxidant system due to the possible change of enzyme activity.

Another possible mechanism of lowering NO bioavailability is inhibiting its production. Thus, we measured plasma NOx concentration which represents endogenous NO production, and found NOx level similar between groups, suggesting an unchanged NO production. To further confirm it, the expression of NOS was also measured. A 43% elevation of eNOS protein expression was detected, but the active form (phospho-eNOS) was not changed. A number of inhibitory effects of HHcy on eNOS activity have been proposed, for example, phosphorylation of eNOS threonine 495 through protein kinase C pathway, and accumulation or activation of endogenous inhibitor of eNOS (Trochu et al. 2003; Jiang et al. 2005; Tyagi et al. 2005). Our microarray also revealed a downregulation of bradykinin type 2 receptor (B2R) by 1.5-fold (*P *=* *0.05). Nonetheless, the NOx level was preserved to control level in HHcy animals, indicating that the impaired NO ability to regulate MVO_2_ is independent of NO production.

Superoxide reacts with NO rapidly and irreversibly to generate peroxynitrite, a reactive nitrogen species. Peroxynitrite can interact with tyrosine residues in proteins to form nitrotyrosine. Although increase in superoxide usually leads to increase of nitrotyrosine, in this study we found the level of nitrotyrosine did not differ between groups. This finding is consistent with a study performed on CBS knockout mice (Powers et al. 2004). While we may conclude an unlikely role for nitrotyrosine as the link between HHcy and CVD in multiparous hearts, exactly how nitrotyrosine remains unchanged remains unknown.

Long-term inhibition of NO synthesis by L-NAME causes cardiac hypertrophy that resembles pressure overload hypertrophy in normal male rats (Sladek et al. 1996). We have demonstrated in this study that NO bioavailability is impaired by increased superoxide in HHcy group. Thus, it is not surprising that the heart weight, LV weight, and HW/BW are all significantly increased as compared to control. In the dilated-hypertrophic heart during pregnancy (Hytten and Paintin 1963; Mabie et al. 1994), NO plays an important role in matching the oxygen supply and demand. Cardiac eNOS expression and NO production are enhanced to inhibit mitochondrial respiration by inhibiting cytochrome c oxidase in cardiomyocytes, resulting in a reduction of oxygen consumption with the same amount of work performed (Wainio 1955; Loke et al. 1999). It is conceivable that mild HHcy limiting the NO availability in the hypertrophic heart of multiparous rats will make the heart more vulnerable and prone to ischemic injury, and increase risk of heart disease later on.

NO can switch substrate utilization in the heart (Suematsu et al. 2007; Williams et al. 2008). It is not surprising that microarray revealed changes in certain metabolism genes, for example, glucokinase regulatory protein and glucokinase regulatory protein. Using large animals with direct access to the coronary circulation will address whether and how substrate utilization is affected in multiparous hearts. Another gene of interest is CREM (downregulated by 1.9-fold), which is an important component in the cAMP signaling network. Its ability to regulate cardiac function and activate NAD(P)H oxidase make it an interesting candidate gene for future study (Muller et al. [Bibr b34],[Bibr b35],[Bibr b36]; Mehrhof et al. 2001; Espinosa et al. 2006; Fu et al. 2006).

In summary, this study for the first time demonstrates that long-term mild HHcy induces hypertension and cardiac hypertrophy, and impairs the ability of NO to regulate cardiac MVO_2_ through NAD(P)H oxidase-derived superoxide production in multiparous mothers. We also provided evidence for how NAD(P)H oxidase is activated, but more specific regulating mechanism that links high tHcy level to the enzyme activation is still needed. Further clarifying the mechanism may have potential therapeutic implications. Microarray has also uncovered certain regulatory genes that might be of pathogenic importance. Mild HHcy alone is able to induce both cardiac functional and genetic changes in multiparous heart, making it vulnerable to any further pathogenetic disturbances. It is reasonable to deduce that mild HHcy is a risk factor for CVD development in pregnancy-related complications such as PE, and at least partially accounts for the increased risk of reoccurrence in later pregnancy.

## Conflict of Interest

None declared.
